# Accelerator Analysis of Tributyltin Adsorbed onto the Surface of a Tributyltin Resistant Marine *Pseudoalteromonas* sp. Cell

**DOI:** 10.3390/ijms9101989

**Published:** 2008-10-24

**Authors:** Haruo Mimura, Ryusei Sato, Yu Sasaki, Yuichi Furuyama, Akira Taniike, Kazutoshi Yoshida, Akira Kitamura

**Affiliations:** 1 Graduate School of Maritime Sciences, Kobe University, 5-1-1, Fukae, Kobe 658-0022, Japan. E-Mails: ryuusei_satou@ulvac.com (R. S.); 088w508w@stu.kobe-u.ac.jp (Y. S.); furuyama@maritime.kobe-u.ac.jp (Y. F.); taniike@maritime.kobe-u.ac.jp (A. T.); kitamura@maritime.kobe-u.ac.jp (A. K.); 2 Hyogo Prefectural Institute of Technology, 3-1-12, Yukihira, Kobe 654-0037, Japan. E-Mail: yoshida@hyogo-kg.go.jp (K. Y.)

**Keywords:** Accelerator analysis, tributyltin, anti-fouling agent, adsorption, cell surface, *Pseudoalteromonas* sp. TBT1, scanning electron microscope

## Abstract

Tributyltin (TBT) released into seawater from ship hulls is a stable marine pollutant and obviously remains in marine environments. We isolated a TBT resistant marine *Pseudoalteromonas* sp. TBT1 from sediment of a ship’s ballast water. The isolate (10^9.3 ± 0.2^ colony-forming units mL^−1^) adsorbed TBT in proportion to the concentrations of TBTCl externally added up to 3 mM, where the number of TBT adsorbed by a single cell was estimated to be 10^8.2^. The value was reduced to about one-fifth when the lysozyme-treated cells were used. The surface of ethanol treated cells became rough, but the capacity of TBT adsorption was the same as that for native cells. These results indicate that the function of the cell surface, rather than that structure, plays an important role to the adsorption of TBT. The adsorption state of TBT seems to be multi-layer when the number of more than 10^6.8^ TBT molecules is adsorbed by a single cell.

## 1. Introduction

Tributyltin (TBT) and triphenyltin (TPhT) chloride were extensively used as anti-fouling agents for the prevention of biofouling on ship hulls since the 1970s. These organotin compounds released from ship hulls resulted in the global distribution in the marine environment [1–7]. Especially, benthic organisms are affected by TBT because the molecule is preferentially adsorbed onto clays and clay-rich sediments [8–10]. For human exposure [7] in general seafood is thought to be the most probable source of TBT [11, 12].

Tributyltin is an environmental endocrine disrupter for aquatic organisms. For example, the induction of imposex, an abnormally progressive formation of a penis and a sperm duct in development of a female, has been observed in abalone [13] and gastropods [14] exposed to TBTCl and TPhTCl. For mammals, TBT showed immunotoxicity in rats [15] and induced apoptosis in rat hepatocytes [16] and rat pheochromocytoma PC12 cells [17, 18]. Organotin compounds are also mutagenic [19]. Therefore, the International Maritime Organization (IMO) prohibited the use of such organotin compounds as antifouling biocides after 1st January 2008, in order to prevent terrestrial and organic pollution [20]. While the efflux of TBT into the marine environment has to be avoided, Lewis *et al*. [21] have pointed out the importance of preventing biofouling on ship hulls for the prevention of the biological invasion of non-indigenous marine organisms into Antarctica. The IMO is enforcing rules regarding discharging ballast water at calls to reduce the potential for transport of non-indigenous species [22]. In addition, restrictions on discharging sediments in ballast tanks seem to be necessary for the near future because they were contaminated with butyltin compounds [23]. The use of natural products, which obtained from marine bacteria on the living hosts of seaweeds and invertebrate, has been proposed for antifouling biocides [24].

Some marine microorganisms which show the resistant ability toward toxicity of TBT have been isolated. An *Alteromonas* sp. M-1 grew in the presence of 125 μM of TBTCl [25], and a protein deduced from the cloned genes responsible for TBT resistance was related to Na^+^/H^+^ antiporters and various Ca^2+^ transporters [26]. An efflux system to pump out TBT from cytoplasm was essential for the TBT resistance of *Alteromonas* sp. [27] and *Pseudomonas stutzeri* [28]. Recently, a TBT-degrading *Aeromonasveronii* was isolated from sediment in an estuarine environment [29].

We isolated a TBT-resistant marine *Pseudoalteromonas* sp. TBT1 from the sediment of a ballast tank of a liquefied natural gas carrier [30]. The isolate was Gram-negative, rod-shaped, and motile and not spore-forming. The partial rDNA sequence of the isolate was deposited in the DDBJ/GenBank/EMBL under the accession number AB298440 [31]. The isolate grew in the presence of 30 μM of TBTCl until the early stationary phase of growth with the same growth rate as that in the absence of TBTCl although the number of colony-forming cells decreased after the cells reached the stationary phase [31]. Kubota *et al*. [32] demonstrated that the total number of the cell-associated Sn atom originated in TBT was counted with an accelerator by a development of the sample preparation method. In this study, we compared the difference of the numbers of the Sn atom originated in TBT adsorbed by lysozyme-treated cells with those by native cells at given concentrations of TBTCl externally added up to 3 mM. Changes in the numbers of Sn atoms originated in TBT and SnCl_2_ were also examined in pH 3.0. We discussed the estimated adsorption state of TBT molecules on the cell surface in relation to an increase in the number of TBT adsorbed by a single cell.

## 2. Results and Discussion

### *2.1. Accelerator Analysis of the Sn Atoms Originated in TBT, or SnCl_2_, Adsorbed by Pseudoalteromonas *sp. *TBT1 Cells*

**Figure 1.**
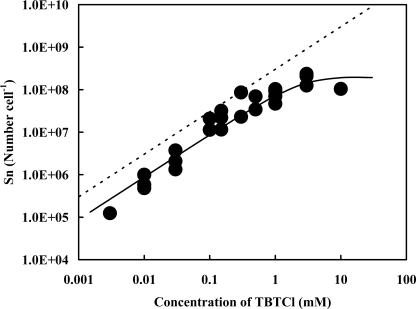
Changes in the total number of the Sn atom originated in TBT adsorbed by resting cells in proportion to the concentrations of TBTCl externally added. A given concentration of TBT was added to the cell suspension (10^9.3 ± 0.20^ CFU mL^−1^) and the number of the Sn atom was measured by the accelerator analysis, PIXE. The averaged experimental error, which is applicable to each data, was estimated to be 41% based on the twenty-five individual data. The dotted line means the calculated maximum number of the Sn atom originated in TBT, which would be available to be adsorbed by a single cell at a given concentration of TBTCl externally added. The solid line shows the regression curve, *y* =1.9×10^8^ (1–exp(−0.45*x*)) (*x* in mM), which was obtained from a total of 25 raw data points in the figure.

Tributyltin chloride, varied from 3 μM to 10 mM as a final concentration, was added into the cell suspension, and the number of the Sn atom originated in TBT adsorbed by the resting cells was measured with the accelerator (Figure 1). The total number of the Sn atom linearly increased with an increase in the concentrations of TBTCl externally added up to 3 mM, where the averaged value was to be 10^8.2^ cell^−1^ (n=2). The value became saturated in the presence of more than 3 mM of TBTCl. Since the isolate could not degrade TBT [31], the entire Sn atoms adsorbed are originated in TBT.

In the presence of less than 3 mM of TBTCl, the numbers of Sn atom actually measured with PIXE were smaller than those calculated under a hypothesis that all of TBT added were adsorbed by the cells suspended in the solution. For example, the number of the Sn atom originated in TBT was actually measured to be 10^7.9 ± 0.13^ cell^−1^ in the presence of 1 mM of TBTCl (n=4). The value on the dotted line should be 10^8.5^ cell^−1^ to the addition of 1 mM of TBTCl externally. This value was about 4 times larger than that obtained experimentally. The difference between the measured and the calculated values was observed for any concentration of TBTCl externally added. The same phenomenon has also been observed for the samples obtained from growing cells [31]. As of now, it is difficult to explain why the values actually measured are smaller than those calculated. A possible explanation is that a certain number of TBT might be desorbed from the cells to reach a new equilibrium condition through the washing process.

**Figure 2.**
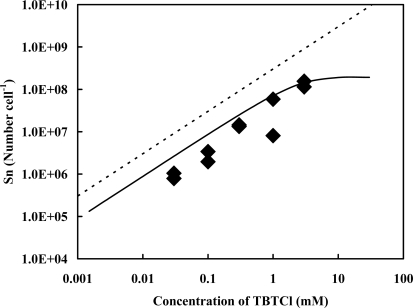
Changes in the total number of the Sn atom originated in TBT adsorbed by lysozyme-treated cells. The dotted and solid lines are the same as those in Figure 1, and the experimental error is 41% for each data.

Figure 2 shows the changes in the total number of the Sn atom originated in TBT adsorbed by lysozyme-treated cells in relation to the concentrations of TBTCl externally added. All the values, except for the one obtained in the presence of 3 mM of TBTCl, were lower than those on the regression curve shown in Figure 1. It might be available to think that the amount of TBT adsorbed by lysozyme-treated cells reduced by about four-fifth comparing with that by native cells.

Since addition of too much lysozyme into the cell suspension and a long period of enzyme reaction with the cells cause cell lysis, we checked the number of colony-forming cells and morphological changes after the cells were treated with lysozyme (1%) for 1 h at 30°C. After the reaction, no reduction of the number of colony-forming cells was observed. As for the morphological changes, cells became spherical shape (Figure 5B) from rod one (Figure 5A). This morphological change indicates the weakness of cell-wall by the lysozyme treatement. The reduction of the numbers of the Sn atom originated in TBT adsorbed by lysozyme-treated cells seems to be closely related to the loss of cell-wall components.

**Figure 3.**
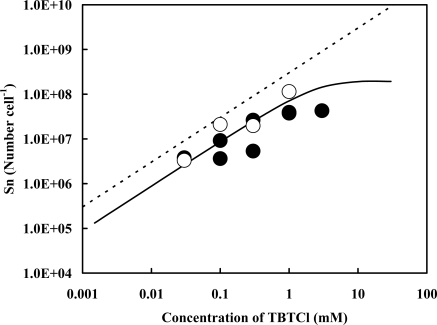
Changes in the total numbers of the Sn atoms originated in TBT (closed circles) and SnCl_2_ (open circles) adsorbed by the cells suspended in pH 3.0. The dotted and solid lines are the same as those in Figure 1, and the experimental error is 41% for each data.

We compared the numbers of Sn atom originated in TBT adsorbed by the cells suspended in pH 3.0 with those in pH 7.8 (Figure 3). Slight reduction of the values in pH 3.0 was observed comparing with those in pH 7.8 on the regression curve shown in the figure. The maximum reduction, 92.1%, was obtained from the value in the presence of 0.3 mM of TBTCl. In acidic pH, a negatively charged cell surface seems to be shifted to a neutral surface because, for example, a carboxylic group in cell-wall components is protonated in the proton rich environment. The surface charge, however, was not affected by an ionic strength of an electrolyte solution [33]. Therefore, the negative charge on the cell surface plays a minor role for the adsorption of TBT^+^ onto the cell surface in pH 7.8.

We also examined the contribution of a hydrophobic interaction of three alkyl groups originated in TBT to the adsorption onto the cells in pH 7.8 by comparing with that of inorganic SnCl_2_ in pH 3.0. When SnCl_2_ was added into the cell suspension adjusted to pH 3.0, the numbers of the Sn atom adsorbed were very close to those on the regression curve obtained in pH 7.8, indicating that the contribution of the hydrophobic nature for those butyl groups is weak or negligible to the adsorption of TBT onto the cell surface.

Hoch *et al.* [34] have examined TBT adsorption onto a mineral, which has ionizable hydroxyl groups on the surface, in pH values varied from 4.0 to 8.0. In this experiment, pH-dependent adsorption of TBT was observed, and the maximum adsorption was found in the range of pH between 6.0 and 7.0. The cation exchange capacity on the surface of the clay was suggested to be closely related to the efficient adsorption of TBT. For the adsorption of TBT by the isolate, *Pseudoalteromonas* sp. TBT1, the number of TBT adsorbed was slightly affected by the exposure to acidic pH, indicating that the isolate has the capacity of cation exchange on the surface of the cells. As another possibility, a receptor protein which has high affinity to a certain substance might recognize Sn atom itself as the substance.

**Figure 4.**
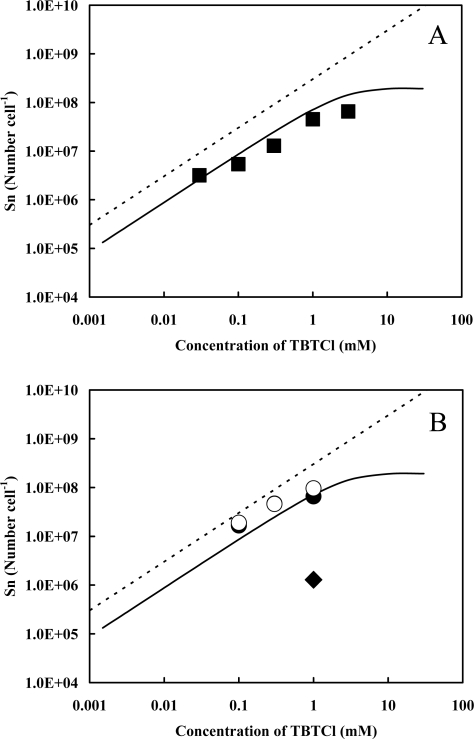
Changes in the numbers of the Sn atom originated in TBT adsorbed by heat-treated cells (A) and ethanol-treated cells (B). As for the ethanol-treated cells, given concentrations of TBTCl were added into the cells (10^9.3 ± 0.2^ mL^−1^) suspended in distilled water (open circles) and the solution which was used for the preparation of resting cells (closed circles). Cells in the solution to which 1 mM of TBTCl was added as a final concentration were washed three times with ethanol (a closed lozenge). The dotted and solid lines are the same as those in Figure 1, and the experimental error is 41% for each data.

**Figure 5.**
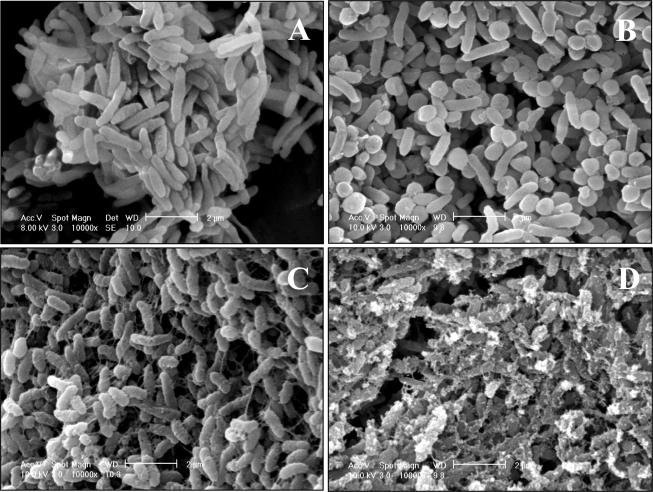
Scanning electron micrographs of the cells. Cells grown until the stationary phase of growth in the medium containing 0.5 M NaCl (A), cells after treated with 1% lysozyme for 1 h at 30°C in the presence of 0.5 M NaCl (B), cells after heated for 1 h at 65°C in the presence of 0.5 M NaCl (C), and cells after treated with ethanol for 1 h at 30°C (D) were observed, respectively, with a scanning electron microscope.

We used heat-treated cells for the adsorption experiment of TBT (Figure 4A). Throughout the given concentrations of TBTCl up to 3 mM, the numbers of the Sn atom adsorbed by the cells were hardly reduced in comparison with those on the regression curve obtained from native cells (see the solid line in Figure 4). As for the morphological changes of the cells, rod shape remained, but new string-like substances were observed (Figure 5C), which look like proteins leached from the cells. In general, heat-treated cells have lost activities of cell-associated proteins. Therefore, it might be concluded that native proteins in the cell do not function to adsorb TBT.

Ethanol-treated cells were exposed to given concentrations of TBTCl when the cells were suspended in distilled water and the solution which was used for the preparation of resting cells (see Experimental Section) (Figure 4B).

Regardless of the suspension medium, the numbers of the Sn atom adsorbed by the cells were hardly changed and those values were close to the ones on the regression curve. In the case of ethanol-treated cells, the membrane structure has been lost because lipids in the cell membrane have been dissolved into ethanol, indicating that the interaction of the cell membrane with TBT does not contribute to an increase in the adsorption of TBT. Rod shape of the cells was damaged and the surface became rough by the treatment of ethanol (Figure 5D). These results indicate that the function of cell surface, rather than the structure of it, plays an important role to the adsorption of TBT. When the cells, which have already adsorbed 10^7.8^ Sn atom cell^−1^ in the presence of 1 mM of TBTCl, were washed three times with ethanol, the value reduced to 10^6.1^ Sn atom cell^−1^, indicating that the affinity of TBT toward the cells is weaker than the solubility of TBT in ethanol.

### 2.2. Estimation of Adsorption State of TBT Based on the Accelerator Analytical Results

**Figure 6.**
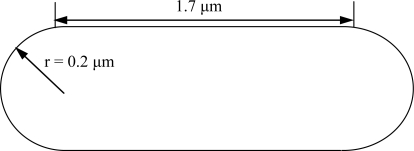
Scheme of rod shape of a cell drawn on the basis of the scanning electron micrograph.

We considered the adsorption structure of TBT adsorbed onto the surface of a single cell of *Pseudoalteromonas* sp. TBT1. We modeled cell shape of the isolate as the combination with two of the half cut of a sphere with the radius of 0.2 μm and a cylinder with the height of 1.7 μm and the bottom area of 0.13 μm^2^ (Figure 6) based on the morphological observation with the scanning electron micrograph (Figure 5A). Following the model, an averaged cell surface area, *S*c, was estimated to be 10^6.4^ nm^2^.

A molecular structure of TBTCl has been simulated using the *ab initio* quantum chemistry package GAMESS [35]. An equivalent radius was estimated to be 0.50 nm based on the empirical molecular orbital calculation software, Scigress Explorer and MOPAC therein (Fujitsu Co., Ltd., Japan). Accordingly, the area occupied by a TBT molecule is plausible to think as the half area of a circle with the radius of 0.50 nm.

If we assume here that the state of TBT adsorption is a single layer on the cell surface regardless of the concentrations of TBTCl externally added, then we can define a virtual area, *S*_o_, which could be equal to an occupational area of a single TBT molecule on the cell surface; *S*_o_ = *S*_c_ / *N*_Sn_. In general, the value of *S*_o_ decreases in relation to an increase in the number of the Sn atom, *N*_Sn_, adsorbed by a single cell. The data on the regression curve in Figure 1 were used as the values of *N*_Sn_ to obtain *S*_o_. As a result, the virtual area varied from 9.5 nm^2^ for the number of 10^5.4^ Sn atom cell^−1^ at 3 μM of TBTCl to 0.013 nm^2^ for the number of 10^8.3^ Sn atom cell^−1^ at 10 mM of TBTCl (Table 1). An appropriate equivalent area of TBT, which was obtained by using computer software, MOPAC, was to be 0.39 nm^2^. The corresponding number of Sn atom adsorbed by a single cell was 10^6.8^ cell^−1^. Since the maximum number of TBT adsorbed by a single cell was experimentally determined to be 10^8.3^ cell^−1^, stacking of TBT molecules onto the TBT-covered cell surface seems to occur at more than 10^6.8^ Sn atom cell^−1^.

Hermosin *et al*. [36] examined the adsorption of monobutyltin (MBT) on clay minerals, and suggested that the MBT adsorption process is a cationic exchange on their surface attracted by electrostatic interaction and an additional adsorption of MBT is the hydrophobic interaction with previously adsorbed MBT by the lipophilic alkyl groups. For the adsorption of TBT by a *Pseudoalteromonas* sp. TBT1 cell, the same adsorption process might be progressed.

## 3. Experimental Section

### 3.1. Isolation of the Pseudoalteromonas sp. TBT1 Strain

A TBT-resistant marine *Pseudoalteromonas* sp. TBT1 was isolate from the sediment in a ballast tank of a liquefied natural gas carrier (110,000 gross tons) returning to Japan from Qatar [30]. The total capacity of the ballast tanks is 55,000 m^3^. On the return trip from Qatar to Japan, almost all the ballast tanks were empty because liquefied natural gas was fully loaded; therefore, the sediment in the ballast tank was available to be sampled. Sediment samples were placed into an autoclaved bottle (250 mL) and kept at 4°C until arrival in Japan. After serial dilution with autoclaved seawater, the sample was spread onto an agar plate containing 5 g Bacto peptone (Difco, MD, USA) per liter of natural seawater, 1 g yeast extract (Difco, MD, USA) per liter of natural seawater, and 150 μM of TBTCl. Incubation was carried out for 5 days at 30°C, and a single colony was picked up and stored at 4°C on the plate.

### 3.2. Preparation of Resting Cells of the Isolate, Pseudoalteromonas sp. TBT1

Cells were grown in a medium containing 5 g Bacto peptone per liter (Difco, MD, USA), 1 g yeast extract per liter (Difco, MD, USA), 0.4 M NaCl, 10 mM KCl, 10 mM CaCl_2_ 2H_2_O, 53 mM MgCl_2_, and 28 mM Na_2_SO_4_. The medium pH was adjusted to 7.8 using tetramethylammonium hydroxide (TMAH). After pre-incubation for one day at 30°C in the medium, incubation was carried out by the addition of a cell suspension to the medium to give a one-thousandth dilution.

Resting cells were prepared from cells grown until the early stationary phase. After 1 mL of cell suspension was harvested by centrifugation (10,000g, 5 min), cells were washed twice with a solution containing 0.4 M NaCl, 10 mM KCl, 10 mM CaCl_2_ 2H_2_O, 53 mM MgCl_2_, and 28 mM Na_2_SO_4_, where the pH was adjusted to 7.8 using 10 mM 2-[4-(2-hydroxyethyl)-1-piperazinyl] ethanesulfonic acid (HEPES) – TMAH. The cells thus obtained were resuspended in 1 mL of the same solution, unless otherwise noticed.

### 3.3. Preparation of the Cells Treated with Lysozyme, Heat, Ethanol Exposure, and Acidic Exposure

Resting cells suspended in the solution described above were treated with lysozyme, which hydrolyzes preferentially the *β*-1,4 glucosidic linkages between *N*-acetylmuramic acid and *N*-acetyl-glucosamine in the mucopeptide cell wall, for 1 h at 30°C prior to the addition of TBTCl at given concentrations. This process did not reduce the number of colony-forming cells. The lysozyme-treated cells were washed twice and resuspended in the solution described above. Then TBTCl was added to give final concentrations of up to 10 mM. After vigorous shaking for 1 min, cells were washed three times with distilled water. The samples thus obtained were stored at 4°C until use.

Acidic exposure to the resting cells was carried out for 1 h at 30° C by the addition of an acidic solution into a cell pellet. The acidic solution contained 50 mM glycine, and pH was adjusted to 3.0 by HCl. Given concentrations of TBTCl, or SnCl_2_, were added into the sell suspension. After vigorous shaking for 1 min, cells were washed three times with distilled water. The samples thus obtained were stored at 4°C until use.

Resting cells suspended in the solution were heated for 1 h at 65°C. After cooling down, TBTCl was added in there to give final concentrations of up to 10 mM. After vigorous shaking for 1 min, cells were washed three times with distilled water. The samples thus obtained were stored at 4°C until use.

Ethanol-treated cells were obtained from the cells exposed to ethanol for 1 h at 30°C. Prior to the addition of TBTCl, the cells were washed twice with distilled water, or the solution described above. After the addition of TBTCl at given concentrations, they were vigorously shaken for 1 min and then washed three times with distilled water. After vigorous shaking for 1 min, the sample in which cells were suspended with the solution in the presence of 1 mM of TBTCl was washed three times with ethanol. The samples thus obtained were stored at 4°C until use.

### 3.4. Enumeration of the Number of Colony-forming Cells

Serial dilution of the sample was carried out with the solution described above after the cells were treated with or without lysozyme. Then cells were spread onto the agar plates containing the same composition as used in the growth medium. Plates were incubated at 30°C for 1 day, and the colonies on the plates were counted. The data are shown as colony-forming units (CFU) per mL.

### 3.5. Accelerator Analysis

Each sample was dropped onto a hollow (2 mm diameter × 0.5 mm depth) in a carbon plate (20 mm × 100 mm × 2 mm thickness) by pipetting and gently dried for about 40 min. Some repetitions of this process were required to finish loading the sample, during which process the volume of the sample was drastically reduced in the hollow [32]. A droplet containing 10 nmol of Sr(NO_3_)_2_ was added into each sample as an internal reference prior to loading the sample onto the hollow.

Quantitative analysis of the Sn atom originated in TBTCl molecule adsorbed by the cells was performed by the analysis of particle-induced X-ray emission (PIXE). The samples were analyzed in a vacuum chamber connected to the M30 beam line of a 5SDH-2 Pelletron accelerator under a pressure of 1.0 × 10^−4^ Pa. The sample target [31] was exposed to a probe beam of 3.0 MeV protons at an incident angle of 0° with a beam current of 2 nA and a beam diameter of 1 mm. Proton incidence of 3.0 μC was necessary to obtain reasonable statistical accuracy of the X-ray yields from Sn and other atomic species measured under the present condition. A Si-PIN photodiode detector was positioned at a 135° angle to the incident direction of the probe beam. A piece of aluminum foil with 90 μm thickness was mounted in front of the detector to reduce low-energy background X-rays and scattered protons. The solid angle subtended by the Si-PIN detector was 5.4 × 10^−2^ sr.

Figures in the text show the relationship between the concentrations of TBTCl externally added to the cell suspension and the amounts of Sn originated in TBT adsorbed by a single cell. Major experimental error in the estimation process of the number of Sn adsorbed by a single cell seems to occur when the value of the number of Sn measured with the accelerator was divided by the number of colony-forming cells used in the experiment because, in general, the colony-counting method described above contains the experimental error of within 10^0.20^ CFU mL^−1^.

### 3.6. Preparation of Samples for Scanning Electron Microscopy

Cells were fixed in 1% (vol/vol) glutaraldehyde for 1 h at 4°C; dehydrated once for 1 h in 50, 70, 90, 95, and 100% of ethanol; and suspended in 100% of *t*-butyl alcohol. After being freeze-dried, they were pasted on a carbon tape and coated with Pt-Pd particles under vacuum. The samples thus obtained were observed with a scanning electron microscope (XL30 CP, FEI Company, Eindhoven, The Netherlands).

### 3.7. Reagents

Tributyltin chloride (95%) and SnCl_2_ (97%) were purchased from Wako (Osaka, Japan) and dissolved in ethanol and in 50 mM of glycine-HCl, pH 3.0, respectively. The latter compound does not dissolve in neutral to alkaline pH. Other chemicals were analytical grade.

## 4. Conclusions

A TBT resistant marine bacterium, *Pseudoalteromonas* sp. TBT1, isolated from sediment in a ship’s ballast tank was used in this experiment. As for the numbers of the Sn atom originated in TBT adsorbed by resting cells, the maximum value experimentally determined was 10^8.3^ cell^−1^. When the cell surface was treated with the enzyme, lysozyme, the amount of TBT adsorbed was reduced. A cell surface charge and a hydrophobic interaction between the cell surface and TBT were not the major factors for the adsorption of TBT by the cells. Heat-treated and ethanol-treated cells also adsorbed almost the same amounts of TBT as those for untreated cells. Throughout the experiments, it is suggested that the function of components on the cell surface, rather than those structure, is important to the adsorption of toxic TBT. The state of TBT adsorption onto a single cell is estimated to be multilayer when the number of TBT adsorbed is more than 10^6.8^. The isolate has a potential application to the recovery of free TBT in seawater because of the high capacity of biosorption of TBT.

## Figures and Tables

**Table 1.** Changes in the estimated virtual area of a TBT molecule when all of TBT adsorbed by a single cell would make a single layer on the cell surface.

TBTCl (mM)	Number of Sn, *N*_Sn_ (Sn cell^−1^)*a*	Virtual area, S_o_ (nm^2^)*b*
0.0030	10^5.4^	9.5
0.010	10^5.9^	2.9
0.030	10^6.4^	0.96
0.10	10^6.9^	0.29
0.15	10^7.1^	0.20
0.30	10^7.4^	0.10
0.50	10^7.6^	0.064
1.0	10^7.8^	0.035
3.0	10^8.2^	0.017
10	10^8.3^	0.013

*^a^* Each data point shown here corresponds to those on the regression curve in Figure 1.

*^b^* The virtual area, *S*_o_, was calculated following the equation: *S*_o_ = *S*_c_ / *N*_Sn_, where *S*_c_ (= 10^6.4^ nm^2^ ) is the surface area of a single cell; and *N*_Sn_ is the number of Sn atom cell^−1^.
